# Closing the micronutrient loop: recovering and redirecting trace elements from food supply chains for a circular and nutrition-secure future

**DOI:** 10.3389/fnut.2026.1912967

**Published:** 2026-08-04

**Authors:** Shan He, Yixiao Wu, Matt Jellicoe, Jiayu Yang

**Affiliations:** 1 School of Food Science and Pharmaceutical Engineering, Nanjing Normal University, Nanjing, Jiangsu Province, China; 2 Faculty of Science, Engineering and Technology, Charles Darwin University, Darwin, NT, Australia; 3 College of Science and Engineering, Flinders Institute for Nanoscale and Technology, Flinders University, Bedford Park, SA, Australia; 4 Bionexus Pty. Ltd., Adelaide, SA, Australia

**Keywords:** biofortification, circular economy, food waste, micronutrient recovery, nutrient recycling, nutrition security, sustainable food systems, trace elements

## Abstract

Micronutrient deficiencies remain a major issue in the lives of billions of people worldwide. Meanwhile, significant losses occur along the food chain for many important micronutrient trace elements, which further compounds this global nutrition problem. The micronutrient stream of iron, zinc, selenium, copper and others are lost in agriculture residues, food processing by-products, retail waste, wastewater and post-consumer organic wastes. These deficiencies do not only lower nutritional efficiency, but also lead to environmental degradation and resource depletion. In response, approaches to the circular economy that focus on nutrient recovery and reuse are coming from the research realm as promising strategies to increase sustainability and nutrition security. This review compares and discusses the micronutrient losses from all stages of the food system and the technologies available to recover trace elements from organic waste streams. Recovery methods such as biological, physicochemical, thermal and hybrid processes are critically discussed with respect to recovery efficiency, scalability and environmental performance. The review also reveals significant safety concerns for nutrient recycling, such as toxic metal contamination, pathogens, pharmaceutical residues, and regulatory constraints. Reintegration options for recovered micronutrients into food systems are also discussed, such as crop biofortification, food fortification, nutraceutical production and animal feed applications. Furthermore, the impact of micronutrient circularity on nutrition, environment and economy is evaluated within the context of sustainable food systems and circular bioeconomy frameworks. The findings of this review confirm that the micronutrient loop can help simultaneously address the food waste, finite resource, and nutrition security challenges globally. But there are considerable technological, regulatory harmonization and consumer acceptance issues that will need to be resolved before this can be widely adopted.

## Graphical Abstract

**Figure:**
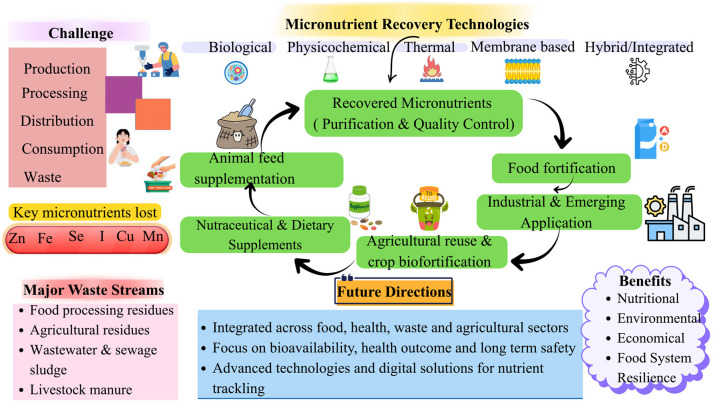

## 1 Introduction

Micronutrients are vital to the growth, metabolic regulation, immune function, cognition and health of humans. In addition, there are trace elements that, although present in very small amounts, are essential for normal physiological functions such as Fe, Zn, Se, I, Cu, Mn, and Mo, that are critical for the body's function (1). Yet deficiencies of these micronutrients are one of the most prevalent types of malnutrition in the world. Hidden hunger affects over 2 billion people, especially in low- and middle-income countries, where eating patterns are rich in energy-dense, nutrient-poor foods (2, 3). The public health and economic burden of IDA, zinc deficiency and low selenium intake persists, influencing maternal health, child development, immunity, and labor productivity (4).

Meanwhile, modern food systems produce vast amounts of nutrient-rich waste. Around one-third of all food produced for human consumption is lost or wasted along food chains every year. These losses occur during crop production, while it is being harvested, processed, transported, sold, consumed at home, and disposed of after use (5). The micronutrient aspect of food waste is not well explored when compared to the other aspects of food waste, namely the calorie loss or economic loss. Sizable losses of key trace elements are occurring in crop residues, fruit and vegetable peelings, processing wastes, livestock manure, waste water sludge and human excrement (6, 7). As a result, these micronutrients, which have taken a lot of land, water, fertilizer, labor and energy to produce, are lost from productive cycles and are frequently discharged into the landfills, into the incinerators or into water bodies (8).

The linear use of nutrients in present food systems has created a significant paradox. The risks of micronutrient deficiencies for human health and nutrition security remain a global concern, whereas vast amounts of micronutrient-enriched biomass are being produced and wasted, with little recovery or reuse (9). An inefficient use of resources is no longer viable in a world with an expanding population, environmental damage, climate change and pressure on limited mineral resources. In conventional food systems, production, consumption and waste management are largely disconnected, resulting in one-way nutrient flows from the agricultural soil to the consumer and then to the waste stream; only a small proportion of nutrients is reentangled in productive systems (10, 11).

The circular economy is a promising approach to combating these challenges. The goal of circular systems is to prevent nutrients from being lost, to use resources as long as possible and to recover valuable substances as much as possible (10). In the past, nutrient recycling mainly focused on macronutrients, including N, P, and K, but the micronutrients and trace elements are gaining more attention in the context of their importance in human nutrition and ecosystem health (12). The recovery of these nutrients from waste streams of the food system has potential for enhancing resource efficiency and reducing hidden hunger and nutrition inequalities.

There has been rapid progress in the development of nutrient recovery technologies with the advancement of biotechnology, environmental engineering, and food processing. The organic wastes, agro-industrial residues, food by-products and wastewater are now considered to be sources of nutrients and not a burden of waste disposal (6). Micro-nutrients recovery technologies like anaerobic digestion, composting, membrane filtration, bioleaching, adsorption, precipitation, hydrothermal processing, and microbial biorefineries have proven their potential to recover valuable micronutrients (13, 14). The recovered nutrients can then be used in closed-loop food systems, such as fertilizers, biofortification, dietary supplements, animal feed, and fortified food products (10).

Micronutrient recovery also provides important environmental benefits. Food waste disposal can result in greenhouse gas emissions, eutrophication, water pollution and soil degradation, while discharges of wastewater can cause excess nutrients and contaminants to be released into ecosystems (15). Recovering nutrients from these streams can reduce environmental impacts, reduce dependence on virgin mineral resources, and enhance resilience to fertilizer market and supply chain disruptions.

The technical, regulatory and social challenges to micronutrient circularity are huge. The extractability of trace elements is complicated by the presence of low levels of elements and their complex chemical associations. Heavy metals, pathogens, pharmaceuticals, micro-plastics and other contaminants can be found in waste streams, which can pose a risk to product safety (16). The recovery of nutrients is therefore essential and the purity, bioavailability and stability of the recovered nutrients are crucial. The regulatory structures are generally not cohesive and consumer acceptance of nutrient recovery from waste derived sources is yet to be determined (17).

Another constraint is the poor linkage between nutrition science and studies on the circular economy. There is little focus on nutritional bioavailability, dietary effectiveness, food fortification compatibility and long-term health effects, with most studies focusing on waste management and agricultural recycling. This review thus explores micronutrient losses throughout food supply chains, outlines the recovery technologies available, considers safety and regulatory aspects, reviews pathways to reintegration into food and agriculture and addresses implications for nutrition security, environmental sustainability and transitions into circular bioeconomy.

## 2 Mapping micronutrient flows and losses in food supply chains

The food supply chains of today are very complex, with nutrients moving from production to waste disposal. In each of these interrelated phases large losses or degradation of valuable micronutrients occurs and micronutrients are lost from the human food chain. Agricultural biomass, processed foods, wastewater flows and organic residues contain trace elements (such as Fe, Zn, Se, I, Cu, Mn) as part of the product (1). Currently, food systems are mostly based on a linear pathway where nutrients are extracted, consumed, then discarded and not recovered (9).

**Figure 1:**
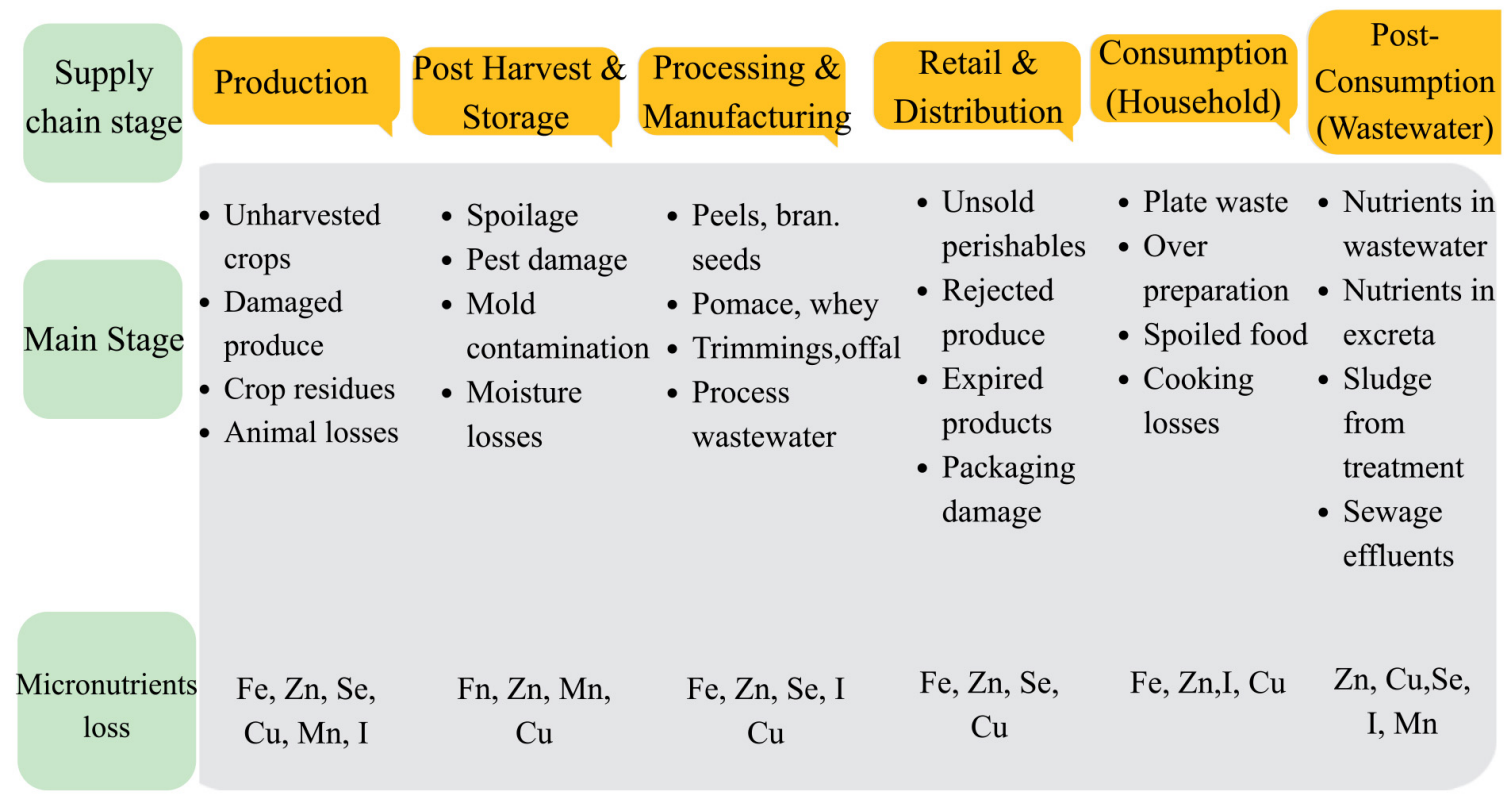
Representation of micronutrient flow, major nutrient loss points, and potential recovery pathways across different stages of the food supply chain within a circular food system framework.

Losses of micronutrients take place at various points and are highly variable by region, food type and chain. In poor areas, losses tend to occur in the production, harvest and storage phase, where infrastructure is lacking and environmental factors are causing stress (18). On the other hand, industrial food systems have higher losses in retailing and household consumption. In addition, the leakage of nutrients to the environment via wastewater and sanitation infrastructure is a significant, but neglected, source of micronutrient loss (19). The knowledge of these nutrient flows is crucial to define the intervention points and design circular recovery systems that will help decrease hidden nutrient losses and increase the sustainability and nutrition security. The main routes of micronutrient flow, loss and recovery potential along food supply chains are shown in Figure 1.

### 2.1 Production-stage losses

Micronutrient leakage in food supply chains is the first big step in agricultural production. Previous research has shown that soil quality, climatic conditions, agronomic management, pest infestations, crop diseases and harvesting practices affect nutrient losses during this phase. Agricultural (or food) soils in many regions, especially in developing countries, are deficient in micronutrients and this can hamper the ability of edible crop tissues to accumulate trace nutrients, including iron, zinc and selenium. Furthermore, the lack of nutrient replenishment during long-term cultivation, also contributes to the loss of soil nutrients and the decline in the nutritional quality of the crops (12, 20).

It was also found that losses due to harvesting and post-harvest operations also play an important role in micronutrient losses. Loss of valuable biomass occurs from mechanical damage, incomplete harvest and reject due to cosmetic criteria. Spoilage, fungal contamination and insect infestation can occur more quickly if the drying and storage conditions are poor and this may affect the nutrient retention and availability in the product (21).

Also, livestock and aquaculture operations have significant nutrient-rich by-products such as blood, bones, organs, fish heads, skins, and viscera that are underutilized (5). In addition to this, agricultural residues like straw, husks, leaves and stems also have high micronutrient recovery potential and are often burnt or left as waste (6). The total of all these losses during production represents a significant loss of micronutrients in linear food systems (Figure 1).

### 2.2 Processing and manufacturing losses

Some of the most concentrated waste streams in food systems are generated in food processing and manufacturing stages, and are rich in micronutrients. Many of the by-products generated during the industrial processing, such as bran, pomace, peels, pulp, husks, whey, spent grains, slaughter residues, contain high levels of trace elements and bioactive compounds (22).

Micronutrient losses are significant, such as the milling away of bran and germ layers that contain the most iron, zinc, selenium and other minerals, resulting in a loss of micronutrient quality from milling. Likewise, the processing of fruits and vegetables creates nutrient-rich peels, seeds and pomace; and additionally, thermal processing may cause a loss of nutrients due to leaching and oxidation (22, 23).

Animal based processing systems also result in losses of micronutrients because the whey and seafood residues, blood and organ tissues that contain valuable minerals from the animal are discarded. Furthermore, the nutrient-rich industrial wastewater from a brewery, dairy or food processing plant is often treated with a primary emphasis on the removal of pollutants, rather than recovery of nutrients (24, 25).

By-products of processing are high in nutrients, and their composition is generally rather homogeneous, which makes them suitable for recovery and reutilization of micronutrients. The value of the micronutrients that are concentrated in the processing by-products also provides key recovery opportunities as shown in Figure 1.

### 2.3 Retail and consumer-stage losses

Food waste and losses of micronutrients occur at retail and household stages, especially in the case of high-income countries (26). Esthetic considerations, inventory control, over-purchasing and consumer food preferences result in the waste of substantial amounts of nutritious food. Small cosmetic flaws are a common reason for product rejection, especially when the product is a micronutrient-dense food like fruit and vegetables, dairy products, and animal-based products (27). Food is wasted because of the discrepancies in demand estimation and overstocking.

Avoidable household food waste is driven by over-purchasing, meal planning issues, misunderstanding of the expiration dates and insufficient storage. The preparation and cooking techniques can also affect the micronutrient retention since undue washing, extended cooking time, and discarded cooking water can result in decreased mineral content and nutritional value (28).

Retail and consumer-oriented losses are large-scale challenges to nutrient-use efficiency in today's food system as shown in Figure 1. Household food waste is highly dispersed, heterogeneous, and is more difficult to recover nutrients from than is industrial by-product food waste. However, there can be improvement in future recovery opportunities in the source separated organic waste collection systems (29, 30).

### 2.4 Post-consumption losses (wastewater and excreta)

In addition to the losses occurring during the processing part of the food system, the post-consumption stages are also significant sources of micronutrient losses (24). Large amounts of micronutrients enter the wastewater and sanitation system after consumption, via human excrement. The concentrations of iron, zinc, selenium, iodine, copper and other trace elements are detectable in urine and feces, but conventional sanitation systems are not focused on the recovery of nutrients (25, 31). Consequently, the presence of valuable micronutrients in wastewater is often lost during wastewater treatment and management of sludge.

Nutrient sources for municipal and industrial wastewater streams include households, food processing plants, hospitals and commercial operations. Trace elements can be left in effluents, deposited in sludge or undergo chemical changes during the treatment process. Sewage sludge can be used directly but the presence of recoverable micronutrients is under concern because of presence of heavy metals, pathogens, pharmaceutical residues and microplastics in the sewage sludge (13).

One nutrient-rich waste source that has been of special interest is human urine, which has significant recovery potential. Nutrient recovery efficiency (ORE) is being explored by developing new technologies such as source-separation systems, membrane filtration, adsorption, ion exchange and electrochemical processes (15, 25, 31). As illustrated in Figure 1, wastewater and sanitation systems offer important opportunities for closing nutrient loops. But there are still a lot of challenges in the form of regulations, safety issues, and public acceptance of the implementation of wastewater nutrient recovery systems (NRS) on a large scale (32).

### 2.5 Quantitative estimates of micronutrient losses across food supply chains

While the amount of wasted food and organic residues are typically calculated in terms of mass or calories, a growing body of evidence indicates the vast amounts of micronutrients in wasted food and organic residues. The losses of nutrients depend on the type of food, geographical region and position in the value chain (33). Perishable, nutrient dense foods like fruit, vegetables, meat, dairy and seafood drive a disproportionately high share of micronutrient loss (34).

Nearly one-third of the world's food is lost or wasted every year, which means that significant amounts of iron, zinc, selenium and other trace elements are lost as well (33–35). Production and post-harvest stages are important nutrient loss locations in developing regions while industrialized countries have retail and household stages. Consumption of micronutrients in wastewater systems also accounts for a loss of nutrients through the sludge and aquatic environment. From a conceptual point of view, micronutrient leakage is ongoing throughout food supply chains, as illustrated in Figure 1, and quantitatively in Table 1.

**Table 1:** Summarizes estimated micronutrient losses and major waste streams across food supply chain stages.

Supply chain stage	Major waste streams	Key micronutrients lost	Estimated contribution to total food loss	Main causes	References
Agricultural production	Crop residues, damaged produce, animal by-products	Fe, Zn, Se, Cu, Mn	15%−25%	Pest damage, harvesting inefficiencies, soil depletion	(81, 82)
Post-harvest and storage	Spoiled grains, fruits, vegetables	Fe, Zn, Mn	10%−20%	Poor storage, microbial spoilage, moisture	(83, 84)
Processing and manufacturing	Bran, peels, whey, pomace, slaughter residues	Fe, Zn, Se, I	20%−30%	Refining, trimming, wastewater discharge	(85, 86)
Retail distribution	Unsold perishables, rejected produce	Fe, Zn, Se	5%−15%	Cosmetic standards, overstocking	(83, 87)
Household consumption	Plate waste, expired foods	Fe, Zn, I	15%−25%	Over-purchasing, poor storage, cooking losses	(82, 83, 88)
Wastewater and excreta	Sewage sludge, urine, effluents	Zn, Cu, Se, I	Difficult to quantify	Sanitation losses, nutrient dilution	(78, 89)

As can be seen in Table 1, micronutrient loss is a continuous process along the whole food supply chain and generates significant losses in the global nutrition systems. These losses can be mapped to identify high value recovery opportunities and the development of circular strategies that can help to redirect nutrients back into food and agricultural systems.

## 3 Recovery technologies: current state and emerging innovations

The losses of valuable micronutrients along the food supply chain are becoming widely recognized, and thus technologies to recover these micronutrients from organic wastes, food-processing residues, wastewater, manure and human excreta are becoming more popular. These strategies cut down on disposal and advocate for resource circularity and nutrient valorization, unlike traditional waste management, which is focused on disposal. These strategies reduce disposal and foster resource circularity and nutrient valorization (10, 30). It is difficult to recover trace elements however due to their low concentrations and they may be linked to contaminants. The recovery technologies used now are: biological, physicochemical, thermal, membrane and hybrid systems.

### 3.1 Biological recovery technologies

The biological recovery approaches involve the use of microorganisms, enzymes, algae, or biological metabolic pathways to mobilize, concentrate, or transform micronutrients from wastes to recoverable forms. They are becoming increasingly attractive due to their low energy consumption, environmental friendliness and the possibility to use them in circular bioeconomy systems.

#### 3.1.1 Composting and vermicomposting

Composting is actually a very old and popular nutrient recovery method. Microbial decomposition process during composting transforms the organic wastes to stabilized humus like material with mineralization and availability of bio nutrients (36). Aerobic composting systems can be used to process food waste, crop residues, manure and agro-industrial by-products. However, as organic matter breaks down and moisture levels decrease, trace elements like iron, zinc, manganese, and copper can be deposited in the final compost product (37).

The earthworms and microbial communities involved in vermicomposting also help to further stabilize the nutrients. Earthworms break down organic substrates, stimulate microbial activity and enhance the efficiency of mineralization. The bioavailability of micronutrients and reduced phytotoxicity in vermicomposted products have been reported to be better than in conventional composts (37, 38). The products can then be applied as soil amendments or biofertilizers in agricultural systems.

However, composting systems can also be contaminated with undesirable substances when feedstocks are contaminated with heavy metals or industrial wastes. Hence, the quality monitoring of feedstocks is still necessary for safe reutilization of micronutrients.

#### 3.1.2 Anaerobic digestion

One of the most widely used technologies for simultaneous waste stabilization, biogas production and nutrient recovery is anaerobic digestion. In this process the microorganisms break down the organic matter in an oxygen-free environment and generate biogas with methane content and digestate as a nutrient-rich digestate. The digestate also contains most micronutrients such as iron (Fe), zinc (Zn), copper (Cu), and manganese (Mn) which will be retained in the digestate after digestion (24, 32).

Digestate can be used as a secondary source of nutrient for crop production after suitable treatment and stabilization. In other systems, further separation and nutrient concentration processes are implemented to further improve nutrient recovery. In addition to nutrient recycling, anaerobic digestion (10) is also beneficial for circular nutrient management with the production of renewable energy and a compelling waste valorization solution.

But digestate quality and composition will be largely determined by the feedstock. The nutrient contents of digestate from food waste are typically higher than the contents of digestate from municipal sludge, but the risk of contamination could also be higher in some cases. Therefore, further purification is needed prior to the safe application of digestate in agriculture or food production (24, 32).

#### 3.1.3 Bioleaching and microbial extraction

Bioleaching refers to the process of solubilization of the metals and minerals from solid waste by microorganisms. Organic acids, chelators, or redox reactions produced by acid-producing bacteria and fungi render trace elements extractable. Bioleaching was mainly developed for the mining and metal extraction industries and has gained wide research interest for the recovery of micronutrients from various food waste ash and industrial biomass sources such as sewage sludge and agriculture residues (24, 39). Chemical leaching is widely used but has some drawbacks, such as the use of large amounts of chemicals, high environmental impact, and poor selectivity for certain elements (40). Microbial extraction has several advantages over conventional chemical leaching, including the ability to use less chemical, less environmental impact and better selectivity for certain elements. It has been shown that fungal species like *Aspergillus niger* and bacterial species like *Acidithiobacillus ferrooxidans* have been effective in the recovery of zinc, copper, iron and manganese with high efficiency (14).

However, bioleaching techniques are frequently less efficient than other physicochemical extraction techniques and careful optimization of the pH, temperature, oxygen concentration and substrate composition may be required. There are also challenges of operating on a large scale, and the composition of the waste is uneven (32).

#### 3.1.4 Algal and microbial biomass systems

The use of microalgae and microbial biomass systems for capturing nutrients from wastewater and industrial effluents is becoming an increasing research interest. Algae can bioaccumulate the trace elements in nutrient rich water streams and also grow a biomass that can be used in biofertilizers, feed additives or bioproducts (14, 25).

There are algal species that have a strong ability to take up selenium, zinc, iron, and copper. Harvested biomass can be further processed to extract high concentrations of micronutrients or feed it to humans and animals as a supplement to nutrition. Additionally, integrated algal systems can enable carbon capture, wastewater purification and valorization of biomass in circular production systems (41).

However, biomass harvesting, drying, and downstream processing are significant challenges for commercial scalability (14).

### 3.2 Physicochemical recovery technologies

Physicochemical methods involve either chemical reaction, adsorption mechanisms, precipitation, ion exchange or electrochemical processes for micronutrient recovery from liquid or solid waste streams. These methods tend to yield quicker recovery and greater selectivity compared to biological methods.

#### 3.2.1 Chemical leaching and solvent extraction

Chemical leaching is a common method used to recover trace elements from solid wastes. This approach involves dissolving micronutrients in liquid phases and separating and purifying them, using acids, alkalis or chelating agents. Common extraction agents are; sulfuric acid, nitric acid, citric acid and ethylenediaminetetraacetic acid (EDTA) (39, 42).

Iron, zinc, selenium, copper can be extracted from food processing wastes, ash, sludge and agri-biomass through chemical extraction. The use of organic acids has been preferred over strong mineral acids due to their more environmentally friendly characteristics and their compatibility with food grade applications (42).

Although good extraction efficiencies are achieved, the chemical leaching methods produce secondary waste streams and may necessitate extensive purification to remove co-extracted contaminants. Other constraints include consumption of chemicals and operational costs (32).

#### 3.2.2 Adsorption and ion exchange

Adsorption technologies are based on natural or artificial materials that have the ability to selectively bind micronutrients from wastewater or liquid waste. Various technologies have been explored, such as activated carbon, biochar, zeolites, chitosan, and engineered nanomaterials, for trace element recovery (15).

This is especially appealing for biochar-based adsorption systems because biochar can be made from agricultural residues and food waste, thus promoting circular resource uses. Both of these materials possess high surface area and functional groups that can bind metal ions (43).

An additional goal of selective improvement is to use resin-based separation of dissolved nutrients in ion exchange systems. These technologies are being used more and more in wastewater treatment plants, with the aim of recovering valuable trace elements prior to discharge. But the regeneration of adsorption materials and the disposal of used resins are still issues of concern in the operation (15, 25).

#### 3.2.3 Precipitation and crystallization

Precipitation techniques remove nutrients by converting them to an insoluble mineral form that can be removed from wastewater streams. While struvite is the standard application of a precipitation system for phosphorus recovery, trace elements such as zinc, iron and manganese can also be precipitated (44).

Precipitation of the desired minerals can be selectively achieved by controlling the pH and applying chemicals. Crystallization technologies may then be used to obtain nutrient products with high nutrient concentrations, which can be used in fertilizers or on other industrial applications (42). They are relatively straightforward and inexpensive methods, but can have lower recovery efficiencies for trace elements at very low concentrations.

### 3.3 Thermal and thermochemical recovery technologies

Thermal technologies involve processes that use heat to concentrate or transform micronutrients out of organic waste streams or to recover micronutrients. The techniques are particularly suitable for processing large amounts of biomass and sludge.

#### 3.3.1 Pyrolysis

Pyrolysis is a process of thermal decomposition in the absence of oxygen to yield biochar, bio-oil, and syngas. Pyrolysis facilitates many trace elements to be concentrated in the biochar fraction. Biochar produced from food waste, manure, and sewage sludge, can thus also be used as an additional micronutrient source for soil use (32, 39).

Pyrolysis not only lowers nutrient concentrations, but also decreases pathogen levels and stabilizes organic contaminants. However, high temperatures could change the bioavailability of nutrients and could produce undesirable compounds depending on process conditions (43).

#### 3.3.2 Incineration and ash recovery

Burning decreases the amount of waste material, and also increases the concentration of mineral material in the combustion ash. The ash can then be used for the chemical or electrochemical extraction of trace elements like zinc, iron and copper (39, 45).

While incineration is an effective method for reducing waste, with high efficiency, it is also prone to some concerns about air emissions, energy consumption and volatilization loss of some nutrients. In addition, there is a possibility that ash is contaminated by toxic metals and that the ash may not be suitable for agricultural reuse unless it is extensively purified (45).

#### 3.3.3 Hydrothermal processing

The new thermochemical processes that process wet biomass under high temperature and pressure are called hydrothermal carbonization and liquefaction. The processes transform the organic wastes into hydrochar and into bio-organic liquid fractions without drying them fully (42).

Food waste and sewage sludge are well suited to hydrothermal since they have a high-water content. This can result in the recovery of a greater proportion of trace elements, reduced waste, and pathogen control (32).

### 3.4 Membrane-based and electrochemical technologies

Advanced membrane and electrochemical systems have been attracting more and more attention because of their high recovery efficiency, selectivity, and integration capabilities with wastewater treatment systems (46).

#### 3.4.1 Membrane filtration

Selective separation of dissolved nutrients from wastewater streams is possible with membrane technologies such as nanofiltration, reverse osmosis, ultrafiltration and electrodialysis. These are especially suitable for micronutrient recovery from liquid effluents from food industries and municipal wastewater treatment plants (MWTPs) (25, 31, 47). RO systems can also extract the dissolved minerals into other fractions that can be used for additional purification. Electrodialysis is also able to selectively transport ions across membranes with the use of an electric gradient (31). Membrane systems are known for their high recovery rates, but membrane fouling, high operating expenses, and energy demands are among the challenges that limit their applications (25).

#### 3.4.2 Bioelectrochemical systems

Bioelectrochemical technologies are comprised of the microbial metabolism and electrochemical reactions for the purpose of nutrient and metal recovery from wastewater. Microbial fuel cells and microbial electrolysis cells can help selectively deposit, migrate ions and concentrate nutrients, while producing energy (25).

While they are still largely at pilot scale, the systems show great potential to integrate nutrient recovery into circular wastewater treatment schemes.

### 3.5 Integrated and hybrid recovery systems

Researchers are more commonly incorporating several recovery technologies into systems that can increase the efficiency of nutrient recovery while reducing environmental impacts. There are hybrid methods, such as anaerobic digestion and membrane filtration, bioleaching and adsorption, and thermal processing and electrochemical recovery (14, 25).

Integrated biorefinery models are particularly promising because they enable the simultaneous recovery of energy, water, organic matter, and micronutrients from a single waste stream. Such systems enable the transition toward full circular food production systems, so that waste is limited and nutrients are continuously recycled (10) (Table 2).

**Table 2:** Major technologies used for micronutrient recovery from food system waste streams, including their operating principles, suitable waste substrates, target trace elements, major advantages, limitations, and representative literature references.

Recovery technology	Operating principle	Suitable waste streams	Major micronutrients recovered	Key advantages	Major limitations	References
Composting	Aerobic microbial decomposition of organic matter into stabilized nutrient-rich compost	Food waste, crop residues, manure, agro-industrial waste	Fe, Zn, Mn, Cu	Low cost, environmentally friendly, improves soil fertility	Slow process, contamination risks from mixed waste	(42, 90)
Vermicomposting	Earthworm-assisted biodegradation and nutrient mineralization	Food waste, agricultural residues, manure	Fe, Zn, Cu, Mn	Enhanced nutrient bioavailability, reduced phytotoxicity	Sensitive to environmental conditions, limited scalability	(42)
Anaerobic digestion	Microbial degradation under oxygen-free conditions producing digestate and biogas	Food waste, sewage sludge, livestock manure	Fe, Zn, Cu, Mn	Simultaneous energy and nutrient recovery	Variable digestate quality, purification often required	(78, 91)
Bioleaching	Microbial solubilization of metals and minerals using organic acids or redox reactions	Sewage sludge, food waste ash, biomass residues	Zn, Cu, Fe, Mn	Reduced chemical use, selective extraction potential	Slow kinetics, process optimization required	(90, 92)
Algal biomass recovery	Uptake and accumulation of dissolved nutrients by microalgae	Wastewater, industrial effluents	Se, Zn, Fe, Cu	Simultaneous wastewater treatment and nutrient capture	High harvesting and processing costs	(93)
Chemical leaching	Dissolution of nutrients using acids, alkalis, or chelating agents	Ash, sludge, food processing residues	Fe, Zn, Se, Cu	High extraction efficiency, rapid recovery	Chemical consumption, secondary waste generation	(78)
Adsorption	Binding of dissolved nutrients onto adsorbent materials such as biochar or activated carbon	Wastewater, industrial effluents	Zn, Cu, Fe	High selectivity, reusable materials possible	Adsorbent regeneration challenges	(42, 94)
Ion exchange	Selective exchange of dissolved ions using resin systems	Municipal and industrial wastewater	Zn, Cu, Se	High recovery efficiency, precise separation	Expensive resins and maintenance	(78)
Precipitation/crystallization	Conversion of dissolved ions into insoluble recoverable mineral forms	Wastewater, digestate	Zn, Fe, Mn	Relatively simple and cost-effective	Reduced efficiency at low concentrations	(91)
Pyrolysis	Thermal decomposition under oxygen-limited conditions producing biochar	Food waste, sludge, crop residues	Fe, Zn, Cu, Mn	Pathogen reduction, nutrient concentration	Possible changes in nutrient bioavailability	(42)
Incineration and ash recovery	Thermal combustion concentrating minerals in ash fractions	Municipal sludge, industrial biomass	Zn, Fe, Cu	Significant waste volume reduction	Air emissions, contamination concerns	(78)
Hydrothermal processing	High-temperature and high-pressure conversion of wet biomass	Sewage sludge, food waste	Fe, Zn, Cu	Suitable for wet biomass, reduced drying requirement	High infrastructure cost	(90)
Membrane filtration	Selective nutrient separation using semi-permeable membranes	Industrial wastewater, municipal effluents	Zn, Cu, Se, Fe	High separation efficiency, scalable	Membrane fouling, energy demand	(95)
Electrodialysis	Ion migration through membranes under electrical gradients	Wastewater and digestate streams	Zn, Cu, Fe	Precise nutrient concentration and recovery	High operational cost	(91)
Bioelectrochemical systems	Combined microbial and electrochemical nutrient recovery	Wastewater, sludge	Fe, Zn, Cu	Simultaneous energy generation and recovery	Mostly pilot-scale applications	(92, 96)
Integrated biorefinery systems	Combination of biological, thermal, and physicochemical technologies	Mixed organic waste streams	Multiple trace elements	Maximized resource recovery and circularity	Technically complex, capital intensive	(42, 90)

## 4 Safety, contaminants, and regulatory hurdles

The effectiveness of micronutrient recovery systems relies on the efficiency of the recovery, as well as the safety of the recovered products. The contaminants in waste streams can also represent valuable nutrients, which require careful product quality assessment, bioavailability and safety. The waste produced in food processing industries in general has less impact on health, whereas wastewater by-products will need more advanced purification, monitoring and regulation to allow the reintegration of these materials into food and agriculture.

### 4.1 Contaminant risks in micronutrient recovery systems

The threat of the co-recovery of contaminants with valuable trace elements is one of the main challenges of nutrient recovery. Toxic materials can be found in waste streams and may build-up during the recovery processes and later be present in food systems if not carefully managed (16, 48).

#### 4.1.1 Heavy metals and toxic elements

Potential toxic elements like lead (Pb), cadmium (Cd), mercury (Hg), arsenic (As), chromium (Cr) and nickel (Ni) are found in many waste streams (1). Some of the contaminants can be from industrial effluents, agrochemicals, urban runoff, domestic products and environmental pollution (48, 49). Several toxic metals have very similar chemical properties to nutritionally important trace elements and can be recovered at the same time when extracting the latter.

For instance, waste streams can be zinc-enriched, which may lead to the presence of cadmium, and iron recovery processes can inadvertently collect lead and arsenic. The presence of such contaminants is extremely dangerous for human health, such as neurological disorders, kidney damage, developmental abnormalities and higher risk of cancer (16). Therefore, contaminant monitoring must become a part of the entire process of nutrient recovery and product development (32).

#### 4.1.2 Pathogenic microorganisms

Biological contamination is significant for waste streams from wastewater, sewage sludge, livestock manure and human excreta. These materials can harbor bacteria, viruses, protozoa and helminths that have the potential to transmit disease (50, 51).

Pathogen reduction is possible by significant factors using technologies like composting, anaerobic digestion, thermal treatment and hydrothermal processing, but the effectiveness of the treatment relies on the operational conditions of the processes and on process control. The failure to adequately treat it can lead to the retention of pathogens and contamination of agricultural soils, crops and food products (32, 36).

#### 4.1.3 Organic contaminants

Emerging contaminants found in waste streams have received increased attention. Conventional treatment methods may not completely remove a wide range of chemicals, including pharmaceuticals, personal care products, hormones, pesticides, antibiotic residues, and persistent organic pollutants (POPs) from the wastewater and sludge (16, 52).

The compounds may disrupt the microbial community of soil, bioaccumulate in plants, and promote antimicrobial resistance (53). Their presence is a significant issue for nutrient recovery systems designed for food and agriculture applications (50).

This is because the analysis technologies are improving, and the detection and management of these contaminants is becoming an increasingly crucial part of risk assessment frameworks (48).

#### 4.1.4 Microplastics and nanomaterials

Recently, a new issue has been identified in the field of circular nutrient management: microplastic contamination. Municipal wastewater treatment plants are known hotspots for micro-plastics from textiles, packages and consumer products. Such particles may be part of sewage sludge and thus could be introduced into agricultural environments via land application (16).

Likewise, engineered nanomaterials have entered into waste streams as a result of their use in industrial or consumer products, and are now found in increasing numbers. However, the potential impacts of these contaminants on the environment and human health are not fully understood, making it important to be cautious in monitoring and managing them in the long term (48).

### 4.2 Quality assurance and purification strategies

Recovered micronutrients need to be carefully purified and quality controlled to make safe nutrient re-use. The goal is to capture as many nutrients as possible and reduce the amount of contaminants to levels that can be used in agriculture, animal feed or food production (32). A variety of purification techniques are used as per the source material and end use. The micronutrients can be selectively separated from undesirable contaminants by different types of precipitation, filtration, adsorption, ion exchange, and solvent extraction processes. Pathogen and dissolved contaminants removal from liquid waste streams is especially effective using advanced membrane technologies (15, 31).

Another advantage of thermal technologies like pyrolysis, incineration and hydrothermal processing is the reduction in microbial contamination and stabilization of organic pollutants. Likewise, composting and anaerobic digestion can help to reduce pathogen levels and stabilize nutrients when properly managed (32, 36).

Quality assurance consists of frequent testing of nutrients composition, organic pollution, microbial contamination and heavy metals. Standardized analytical procedures are fundamental to ascertain product safety and to make sure product consistency between different production batches. Traceability systems are also gaining significance in recovering information on the origin of feedstocks and their treatment process and quality at various stages of the recovery process (17).

Another important consideration is nutrient bioavailability. Any nutrient content in the recovered products should be left in a form that can be efficiently used by plants, animals and humans. Although nutrients are normally high in recovery efficiency, in some cases the aggressive extraction or thermal treatments can decrease the availability of these nutrients. Thus, it is important to consider the functionality of the food as well as its contaminant removal capabilities (43).

### 4.3 Regulatory frameworks governing nutrient recovery

There are numerous regulatory frameworks in place, and the nutrient recovery regulations differ significantly from country to country and region to region. Current regulations have traditionally been concerned with waste management and protecting the environment, not nutrient circularity and food system reintegration (17).

In the European Union, the focus has been intensified on policy attention for nutrient recycling strategies and circular economy policies. The EU Fertilizing Products Regulation has established mechanisms for putting some recovered nutrient products on agricultural markets, if they are safe and of good quality. Regulations for applications in food and feed are still relatively restrictive, however, for recovered micronutrient applications (54).

Nutrient recovery products are currently primarily regulated in the United States under existing laws and regulations for fertilizers, biosolids, animal feeds and food ingredients. The Environmental Protection Agency (EPA), Food and Drug Administration (FDA) and state regulatory agencies are all responsible for ensuring product safety. There are comparable multi-agency strategies in other countries (55).

One of the greatest obstacles is the lack of internationally aligned standards for micronutrients recovered. The thresholds, testing requirements, labeling rules, and approval procedures for contaminants vary, hindering commercialization and international trade. In addition, regulations often prioritize traditional pollutants and lack clarity on emerging pollutants like pharmaceutical residues, microplastics, and nanomaterials (56).

Nutrient circularity in the future needs to be extended by shifting from waste-based classification toward a resource-oriented system where recovered nutrients are considered as valuable second raw materials. These should be science-based, risk-proportionate and flexible to technological innovation (17, 55).

### 4.4 Public acceptance and social considerations

Although recovered micronutrients can be technically safe and compliant with regulations, the market success depends on the public acceptance. The source of recovered nutrients may affect consumer perception, especially if they are wastewater, sewage sludge or human excreta (55).

The so-called “yuck factor” psychological factors can create resistance to products from waste streams, even if they are proven to be safe (57). The same concerns have plagued recycling water, biosolids and alternative protein sources in the past. Therefore, adequate communication and public awareness are important elements of successful nutrient recovery programmes (17).

Promoting scientific evidence, environmental values and safety verification in education can help to raise public confidence. Consumers may be further reassured by certification schemes, quality labels and third party verification. Results of water reuse and organic waste recycling projects indicate that when the decision-making process involves stakeholders and the benefits are clearly explained, acceptance is greater (55).

Importantly, there is cultural and regional variation in public attitudes, as well as differences in attitudes according to type of application. It may be easier to take in nutrients recovered and incorporated in fertilizers than to eat nutrients that are added directly to food. In addition, social, cultural and behavioral factors need to be taken into account alongside technological and regulatory factors within market development strategies (17).

## 5 Reintegration pathways for recovered micronutrients into food systems

**Figure 2:**
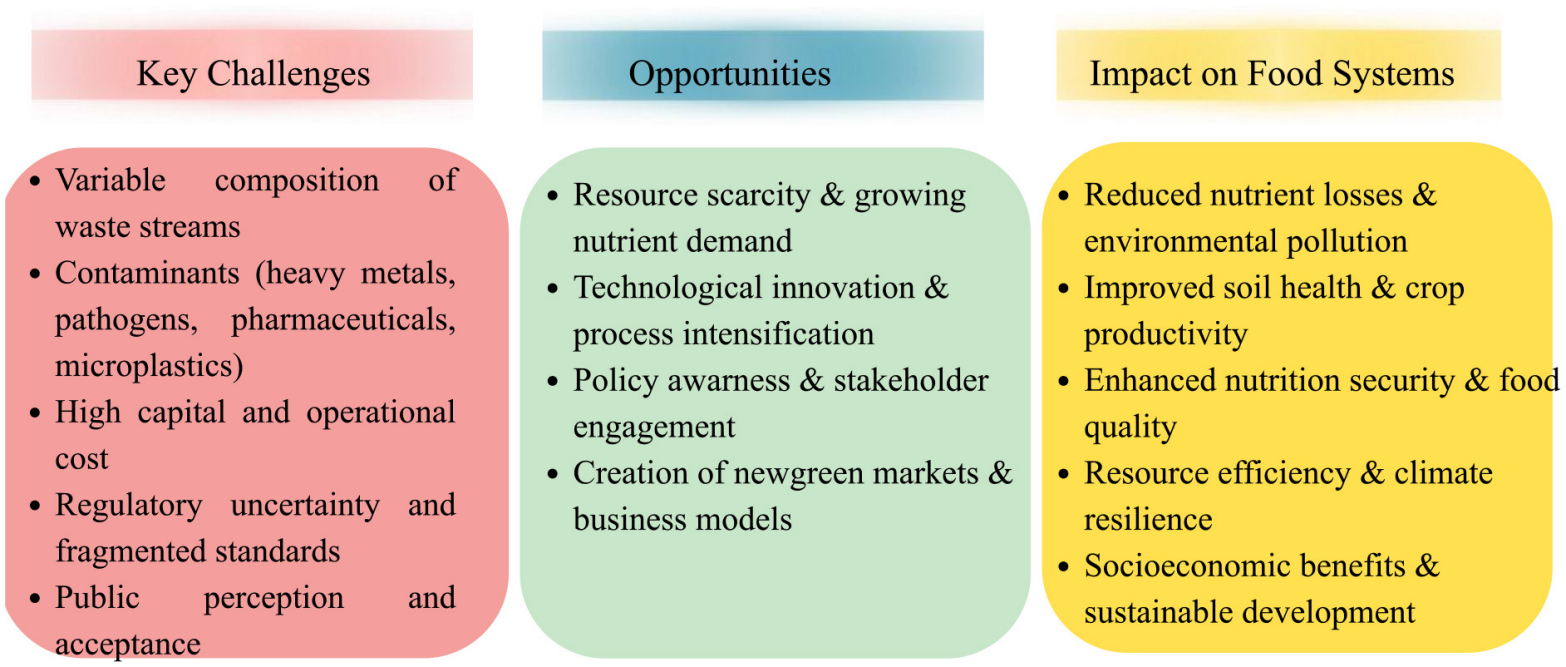
Conceptual framework illustrating the key challenges, enabling drivers, and food-system impacts of micronutrient recovery within a circular bioeconomy.

The main objective of micronutrient recovery is the safe reintegration of recovered nutrients into food systems for the conservation of the resources, sustainability of agriculture, and nutrition security. Trace elements recovered, such as iron, zinc, selenium, iodine, copper and manganese, can be used in agriculture, food fortification, animal feed, nutraceuticals and other approved applications, such as agriculture biofortification. Trace elements recovered, including iron, zinc, selenium, iodine, copper and manganese, can be used in agriculture biofortification, food fortification, animal feed supplementation, nutraceuticals production and others approved applications. These pathways work together to create the micronutrient loop in circular food systems (Figure 2).

### 5.1 Agricultural reuse and crop biofortification

The recovered micronutrients are also important for agriculture, as soil health has a direct impact on food quality and nutritional outcomes. In many parts of the world, the soil is depleted of essential micronutrients (e.g., trace minerals) due to intensive cultivation, erosion, and insufficient nutrient replenishment, resulting in lower micronutrient content of crops and widespread hidden hunger (58, 59). The micronutrients can be re-introduced in the agricultural systems as biofertilizers, composts, digestates, biochar and micronutrient enriched fertilizers. The amendments not only help replenish the soil nutrient stocks but can also enhance soil fertility, organic matter content, water holding capacity and microbial activity (32).

In particular, a promising approach is the agronomic biofortification, in which micronutrients recovered from soil or crops can be added back to soil or crops to boost the levels of nutrients in edible plant parts. The successful ones include biofortification of cereal crops (particularly zinc and selenium) and legumes and vegetables (particularly iron) (58, 60). While the nutrient availability, soil properties and crop species influence the effectiveness, agriculture is one of the most direct routes to better nutrition security via micronutrient recovery.

### 5.2 Food fortification and functional food development

Recovered micronutrients are important tools that can be used to enhance dietary micronutrient intakes and mitigate the risk of micronutrient deficiencies, through food fortification. As purification technologies have improved, opportunities for using recovered Fe, Zn, Se and iodine in food grade products have emerged, but with strict safety and quality standards (9, 32). These micronutrients could be incorporated into staple foods like flour, rice, dairy products, drinks, and complementary foods, helping to improve nutrient supply chains and have the potential to be more sustainable.

Recovered micronutrient could also be used to develop functional foods and value-added ingredients. Fruit pomace, seafood waste, and cereal by-products are rich in minerals that have been leveraged for fortification in food products, drinks, and nutritional supplements (9, 23). But it requires to achieve nutrient stability and bioavailability, sensory acceptability, and consumer confidence for successful implementation. Furthermore, there are strict regulations for direct food use. Despite the difficulties, food fortification presents promise to reintegrate recovered micronutrients into food systems and improve nutrition security (61).

### 5.3 Animal feed supplementation

Animal production systems are an important means of re-integrating recovered micronutrients into food systems. Trace elements include zinc, copper, selenium, iron, and manganese that are needed for livestock and aquaculture to grow, breed, maintain good health and be productive. Nutrient-rich food-processing residues such as microbial biomass, algal systems and other waste streams can potentially be used to substitute for some of the mineral supplements in animal feeds, where micronutrients are recovered. This not only aids in optimizing the use of resources but also helps curb waste production and minimize reliance on raw mineral resources (9, 10).

Indirect nutrition benefits for humans can also arise from feed supplementation, in the form of improved micronutrient profile of animal-derived foods. For instance, selenium in selenium-enriched feeds has been found to raise the selenium level in meat, milk, eggs and fish and similar effects have been observed with zinc and other trace elements (62). However, the recovery of micronutrients for use in feed must be carefully controlled in order to limit the risk of contamination to animal products. Thus, in order to ensure sustainable implementation, it is important to conduct comprehensive safety assessment and monitoring (32).

### 5.4 Nutraceuticals and dietary supplements

There are other opportunities for recovered micronutrient use in the fast-growing nutraceutical industry. Nutraceuticals exist somewhere in the middle between food and pharmaceuticals, and are becoming more popular for treating nutritional deficiencies and promoting health (63, 64). There are numerous waste streams in the food system that are rich in bioavailable micronutrients that can be converted into nutraceutical products of high value. They may include mineralized extracts of fish processing waste, shellfish processing waste, fruit pomace, algae and fermentation residues. Recovery technologies for the targeting and concentration of specific micronutrients, which can then be used for incorporation into capsules, tablets, powders and functional formulations (9, 23).

Nutraceutical products can yield higher value of recovered nutrient per unit production compared to bulk agricultural products. This can help make nutrient recovery systems more financially viable and motivate investors to invest in higher recovery technologies (23).

Nutraceutical applications, however, necessitate strict quality assurance and toxicological testing as well as adherence to food and pharmaceutical regulations. Clinical evidence with long-term efficacy and bioavailability would be required for general acceptance in the market (65).

### 5.5 Industrial and emerging applications

Micronutrients recovered can be applied to a range of industrial applications as well as direct applications in food and agriculture. Trace elements collected from the waste stream can be used for the manufacturing of fertilizers, specialty chemicals, microbial growth media, and bio-based products. These applications do not necessarily have a direct link to human nutrition, but they can enhance the efficiency of resources and boost the economic feasibility of nutrient recovery systems (NRS) (10, 25).

Innovations are also beginning to emerge, creating new potential uses. Precision agriculture, controlled-environment farming, and microbial biotechnology are some of the emerging technologies that could facilitate more precise use of recovered micronutrients (66). The concept of circular biorefineries (CBRs) is increasingly becoming popular, where nutrients, energy, water, and valuable biomolecules are recovered from waste streams in an integrated manner (14, 67).

Better nutrient tracking and allocation across supply chains could be achieved with additional use of digital technologies, artificial intelligence and real-time monitoring systems. These developments could support better alignment of recovered nutrient products and the varied nutritional, agricultural and industrial requirements (68).

### 5.6 Challenges to effective reintegration

Although there is a great potential for the use of micronutrient reintegration pathways, multiple challenges still constrain the broader implementation of these pathways. The nutrient consistency, quality and safety in the waste stream can be influenced by different compositions of the waste stream, sometimes requiring extensive purification before the recovered nutrients can be used in food or feed applications. Additional factors such as the high capital costs, operational expenses and transportation needs could also limit implementation (32, 55).

The bioavailable fraction is also important and the recovery processes should maintain or improve the nutritional efficiency of trace elements without just focusing on maximizing recovery efficiency. Furthermore, the acceptance by the consumers of the waste-derived products and compliance to the regulatory requirements are still challenges (17, 43).

Improved coordination between waste management systems and agricultural sectors, as well as between food industries, public health agencies, and policy makers is needed for effective reintegration. Agricultural biofortification, food fortification, feed supplementation and nutraceutical applications are the important pathways for the reutilization of recovered micronutrients, thus advancing circular food systems, resource efficiency and nutrition security as illustrated in Figure 2 (10, 55).

## 6 Nutritional, environmental, and economic impact assessment

### 6.1 Nutritional implications

The potential contribution to nutrition security is one of the most important benefits of micronutrient recovery. Iron, zinc, selenium, iodine and other trace element deficiencies still continue to impact millions and millions of people across the globe, especially in low income settings. These deficiencies have been linked to developmental delays, compromised immune system, decreased productivity, and vulnerability to illness (28, 69).

Recovered micronutrients may be utilized in several ways to improve dietary intake, such as crop biofortification, fortification of food products, dietary supplements for the animals, and production of nutraceutical (70). Circular recovery systems create an opportunity to recover nutrients from food supply chains or from within food value chains, which is different from traditional methods that are based on mining and industrial production of micronutrient compounds. Therefore, nutrient recovery can be used in addition to current nutrition interventions to enhance the overall use of sources (55, 61).

In particular, the use of recovered micronutrients for agronomic biofortification, which is used to solve nutrient deficiency at the production stage, is more promising. Likewise, fortification of foods and dietary supplements with reclaimed trace elements can enhance micronutrient status of vulnerable population. While more research is required to assess the bioavailability and long-term health effects of recovered micronutrients, current studies indicate that they can be a valuable source to tackle hidden hunger when appropriate safety and quality measures are observed.

### 6.2 Environmental benefits

A significant amount of waste is generated in the food system, impacting the environment. Organic waste will release greenhouse gases (such as methane) into landfills and nutrient leakage will lead to water pollution, eutrophication, and degradation of soil. Meanwhile, the mining and refining of natural resources is also a high-energy process and can have substantial ecological consequences (71).

The recovery of micronutrients contributes to environmental sustainability through nutrient minimization and circularity of resources. The recovery of trace elements from food waste, agricultural residues, wastewater and processing by-products reduces the demand for primary raw materials and reduces the negative impact of waste disposal. Moreover, nutrient recovery can help to lower pollutant loads flowing into aquatic ecosystems and enhance the efficiency of wastewater treatment systems (10).

Reused nutrients when used in agriculture can also help to enhance soil health. Organic nutrient recovery products like composts, digestates, and biochar not only deliver micronutrients, but also improve soil structure, water holding capacity and microbial activity. These benefits can help enhance agricultural resilience in the face of climate changes (43).

Life cycle assessment research has grown in number and frequency, and many show that nutrient recovery systems can have a lower footprint than traditional waste management and nutrient production systems. But, technology and the use of energy, transportation needs and contaminant management are all factors that affect environmental performance. Hence, full-scale evaluations are required to determine the most viable recovery solutions (72).

### 6.3 Economic opportunities and challenges

Micronutrient recovery can be an economic opportunity to convert waste streams from liabilities to assets from an economic standpoint. Nutrients from food processing wastes, wastewater effluents and agricultural residues can be converted to marketable products, providing new revenue streams within circular bioeconomy structures (10, 67).

Recovered micronutrients have the potential to be used in developing a variety of value-added products such as biofertilizers, fortified foods, feed additives, nutraceuticals and specialty chemical products. Diversification can boost the competitiveness of the food processing sector and waste management systems and promotes innovation, creating jobs. Additionally, circular nutrient systems can lessen reliance on external inputs like mineral fertilizers and supplements, enhancing resource security and supply chain resilience (73).

Although these opportunities exist, there are a number of economic hurdles to be overcome. There are significant investment, infrastructure and operational costs associated with many nutrient recovery technologies. The economic feasibility is affected by recovery efficiency, product quality, transportation logistics and market demand. Sometimes nutrient products recovered from waste can face competition with conventional products as lower production costs are associated with conventional supply chains (55).

Therefore, policy incentives, regulatory support and market development mechanisms will be important in encouraging commercial uptake. The circular nutrient systems will become increasingly economically competitive as the technologies for nutrient recovery become more mature, and as economies of scale improve (72).

### 6.4 Contribution to sustainable food systems

The micronutrient loop is closely linked to food system transformation, which is a key objective of sustainable food systems. Circular nutrient management can help achieve several Sustainable Development Goals (SDGs), such as Zero Hunger (SDG 2), Good Health and Well-being (SDG 3), Clean Water and Sanitation (SDG 6), Responsible Consumption and Production (SDG 12), and Climate Action (SDG 13) (72, 74).

Micronutrient recovery establishes synergies between waste valorization and nutrition outcomes, linking the two together. Circular approaches do not only consider food waste as a disposal issue but as a source of valuable nutrients that can be used for the production of food and nutrition in the future (10).

Moreover, circular micronutrient systems help to strengthen resilience by minimizing reliance on limited mineral resources and fragile global supply chains. This is especially relevant given the growing scarcity of resources, geopolitical tensions and climate change impacts. Nutrient recovery can thus play a role in long-term sustainability, resource security and nutritional equity in food system planning (68, 75).

## 7 Policy integration and future directions for circular micronutrient management

Supportive policy and governance frameworks are needed alongside technological advances for transition to circular micronutrient management. Although the nutrient recovery benefits are becoming more widely recognized, the deployment of nutrient recovery technologies is hampered by the lack of a coordinated nutrient management approach across the food, agriculture, waste management and public health sectors, as well as by economic and regulatory obstacles. Effective development of integrated policies that facilitate nutrient recovery while maintaining food safety, environmental protection and nutritional effectiveness will be key for building sustainable and resource-efficient food systems.

### 7.1 Integrating micronutrient recovery into circular economy policies

In order to increase the use of resources and decrease waste, there are strategies being followed around the world, known as circular economy strategies. Most policy initiatives have been, however, mainly dedicated to energy recovery, recycling and macronutrient management, especially N and P. The importance of micronutrients for ecosystem functioning and human health is generally undervalued (55, 72).

To integrate micronutrient recovery in circular economy policies, the food system waste streams should be recognized as secondary nutrient resources instead of disposal burdens. This would stimulate investment in nutrient recovery plants and market development of nutrient products recovered. Policies can be developed that make micronutrient circularity possible, such as policies supporting waste valorization, industrial symbiosis and circular bioeconomy development (68, 76).

Economic incentives, including subsidies, tax relief, green procurement schemes and funding for research and development to promote technology development and commercialization are also effective ways for governments to support implementation. These measures may be used to address the financial challenges that constrain the competitiveness of products containing recovered nutrients compared with traditional products (73).

### 7.2 Regulatory harmonization and quality standards

Lack of harmonized regulatory frameworks is one of the key challenges to micronutrient recovery. Each country has its own standards for waste classification, contaminant levels, nutrient re-use and product approval. Inconsistencies lead to uncertainty for technology developers, investors and end users (55, 77).

Regulatory structures should incorporate more risk-based systems that take into consideration the nutrient produced, not the source from which the nutrients are recovered, and determine if that product can be used as a nutrient in agriculture. Standards for the monitoring of contaminants, nutrient composition, bioavailability, traceability and labeling are needed to assure product safety and market confidence (17).

Special consideration should be placed on emerging contaminants like pharmaceutical residues, micro-plastics, antibiotic resistance genes and engineered nanomaterials. Regulatory systems should be adjusted in light of scientific information continually evolving. To establish standards that are internationally recognized and promote trade and technology transfer, international collaboration will be necessary (16, 50).

### 7.3 Research priorities and knowledge gaps

Progress in nutrient recovery research has been great but there are still a number of gaps in knowledge that need to be addressed. While many of the studies conducted are focused on recovering efficiency and engineering performance, few studies are dedicated to nutritional outcomes and impacts on human health (55, 78).

The bioavailability of recovered micronutrients, long-term agronomic performance, environmental risk assessment and life-cycle sustainability evaluation should be the priority of future research. Further comparative studies for testing various technologies under real-world conditions would be beneficial, especially to determine the most effective and cost-efficient technologies (44, 72).

All this will require interdisciplinary work among nutrition scientists, agronomists, environmental engineers, economists and nutrition policy makers. In addition, there is a lack of better nutrient flow accounting and monitoring systems to measure micronutrient loss and recovery potential along food supply chains (75).

The key policy actions and research priorities needed to progress micronutrient management toward being a circular process are outlined in Table 3.

**Table 3:** Key policy actions, research priorities, expected outcomes, and representative literature supporting the transition toward circular micronutrient management within sustainable food systems.

Priority area	Key actions	Expected outcomes	References
Circular economy integration	Incorporate micronutrient recovery into national circular economy and bioeconomy strategies	Increased resource efficiency and nutrient circularity	(55, 97)
Regulatory harmonization	Develop standardized safety, quality, and contaminant guidelines for recovered nutrients	Improved market confidence and technology adoption	(78)
Food safety monitoring	Establish protocols for monitoring heavy metals, pathogens, pharmaceuticals, and microplastics	Enhanced public health protection	(98, 99)
Bioavailability research	Evaluate plant uptake and human absorption of recovered micronutrients	Improved nutritional effectiveness	(100)
Technology scale-up	Support pilot and commercial-scale demonstration projects	Reduced implementation costs and improved feasibility	(90)
Life cycle assessment	Conduct environmental and economic sustainability evaluations	Evidence-based technology selection	(55, 101)
Stakeholder engagement	Increase public awareness and industry participation	Greater social acceptance and market development	(102)

As shown in Table 3, advancing micronutrient circularity requires coordinated efforts across policy, research, regulatory, and societal dimensions. Technological progress alone will not be sufficient unless accompanied by supportive governance frameworks and stakeholder engagement.

### 7.4 Toward a nutrition-sensitive circular bioeconomy

There is a challenge for future food systems to meet both nutritional needs and environmental sustainability with a focus on resource efficiency. Circular micronutrient management presents a unique opportunity to connect these goals through the linking of waste valorization and nutrition-sensitive interventions. Circular systems aim to provide nutrients to be kept in productive cycles for the longest time possible as opposed to traditional waste management systems which focus on disposal (68, 72).

A nutrition-sensitive circular bioeconomy is not only about recovery of resources but also about the contribution of resources recovered to the well-being of humans. This approach acknowledges that food system sustainability should be measured by the nutritional results of the food system as well as the environmental performance. Including nutrition indicators in the policy framework of circular economy could then enhance the link between sustainability and public health goals (61, 79).

To realize the transition to circular micronutrient management with policy frameworks, harmonized rules, targeted investments in research and active engagement of stakeholders are needed. Incorporating micronutrient recovery into waste valorization could revolutionize waste streams in the food system, providing valuable nutrients for sustainable farming, environmental conservation, and human well-being (80). Future work needs to be directed toward creating nutrition-sensitive circular bioeconomy models which provide the ability to close nutrient loops, while at the same time being safe, economical and socially accepted (55, 75).

## 8 Conclusion

The problem of micronutrient deficiencies and food system inefficiencies are closely related issues that continue to pose a threat to nutrition security and environmental sustainability at the global level. Hidden hunger is present all over the world, but there is a very high prevalence of essential trace elements, such as iron, zinc, selenium, iodine, copper, and manganese, being lost along food supply chains across production, processing, distribution, consumption and waste disposal. These losses decrease nutrient use efficiency and result in the loss of valuable natural resources.

This review shows that food waste, agricultural residues, processing by-products or wastewater and other organic wastes are significant secondary sources of micronutrients. New developments in biological, physicochemical, thermal, membrane-based and hybrid technologies have opened up possibilities to recover and reutilize these nutrients in circular food systems. Agricultural biofortification, food fortification, animal feed fortification or the use of nutraceuticals are useful ways of reintegrating recovered micronutrients.

Beyond the nutrient recovery benefits, the application of micronutrient recovery can also lower the amount of waste generated, offset environmental contamination, and lower the demand for finite mineral inputs. Still, there are issues that must be addressed, including contamination concerns, regulatory obstacles, economic viability and public opinion. Innovation in technology, coordinated policies and interdisciplinary partnerships will be needed to overcome these constraints. Overall, closing the micronutrient loop represents a potential pathway to building more resource efficient, resilient, and nutrition secure food systems.
